# *FLT3* targeting in the modern era: from clonal selection to combination therapies

**DOI:** 10.1007/s12185-023-03681-0

**Published:** 2023-12-19

**Authors:** Vanessa E. Kennedy, Catherine C. Smith

**Affiliations:** 1 Division of Hematology/Oncology, Department of Medicine, University of California San Francisco, 505 Parnassus Ave, Box 1270, San Francisco, CA 94143 USA; 2 Helen Diller Comprehensive Cancer Center, University of California San Francisco, San Francisco, CA USA

**Keywords:** Acute Myeloid Leukemia, AML, FLT3, FLT3 inhibitor, Clonal Evolution

## Abstract

*Fms-like tyrosine kinase 3 (FLT3)* is the most frequently mutated gene in acute myeloid leukemia (AML). Modern targeting of FLT3 with inhibitors has improved clinical outcomes and FLT3 inhibitors have been incorporated into the treatment of AML in all phases of the disease, including the upfront, relapsed/refractory and maintenance settings. This review will discuss the current understanding of FLT3 biology, the clinical use of FLT3 inhibitors, resistance mechanisms and emerging combination treatment strategies.

## Introduction

Acute Myeloid Leukemia (AML) is an aggressive hematologic malignancy with a heterogenous genetic landscape [1]. *Fms-like tyrosine kinase 3 (FLT3)* is the most frequently mutated gene in AML, with approximately 30% of newly diagnosed AML patients harboring an *FLT3* mutation, and *FLT3* mutations are associated with aggressive disease biology [2]. Since *FLT3* mutations in AML were first described over 25 years ago, our understanding of the biology, clonal dynamics, targetability, and prognostic significance of *FLT3* has evolved. Worldwide, there are now 3 approved FLT3 inhibitors, which have been studied as single agents, in combination with cytotoxic chemotherapy, and more recently in combination with other targeted agents. Paralleling progress in FLT3 targeting, the prognosis for patients with *FLT3-*mutated AML has improved in tandem, and as of 2022, high *FLT3* allele burden is no longer considered high-risk in AML [3]. Despite these advances, heterogenous resistance mechanisms to FLT3 inhibitors represent an ongoing challenge and multiple questions regarding *FLT3* in AML remain. In this review, we highlight several recent advances in the understanding of *FLT3* biology, clonal selection, therapeutic targeting, and resistance.

## FLT3 mutational landscape: where are we today?

The *FLT3* gene encodes FLT3, a membrane-bound protein and member of the receptor tyrosine kinase family [4]. Upon binding to the FLT3 ligand, FLT3 undergoes homodimerization and activation, leading to increased cellular signaling via multiple pathways, including RAS/MAPK, JAK/STAT, and PI3K/AKT [4]. Together, this increased signaling promotes cellular proliferation, inhibition of apoptosis, and inhibition of differentiation [5]. FLT3 is widely expressed on normal hematopoietic progenitor cells and is overexpressed on the majority of AML blasts [5]. In normal hematopoiesis, FLT3 activation is tightly regulated via phosphorylation of the protein’s juxtamembrane domain (JMD). When mutations develop in the JMD or adjacent tyrosine kinase domain (TKD), this tight regulation is disrupted, causing constitutive activation, ligand-independent signaling, and clonal proliferation [6].

### FLT3-ITD and TKD

The *FLT3* internal tandem duplication* (*ITD) is the most clinically significant *FLT3* mutation, and patients with *FLT3*-ITD mutated AML have increased rates of disease relapse, and inferior overall survival [7]. Present in approximately 25% of newly diagnosed patients, *FLT3*-ITD mutations occur within the JMD and are variable in size, ranging from 3 to > 1,000 nucleotides [6]. The less common TKD mutations, present in approximately 5% of patients, are missense point mutations in the activation loop of *FLT3*, most commonly at D835. Like *FLT3-*ITD mutations, *FLT3-*TKD stabilizes the active kinase formation and result in constitutive receptor activation [5]. Unlike *FLT3*-ITD mutations, *FLT3*-TKD have an unclear impact on patient prognosis and are not currently included in consensus risk assessments [3, 8].

### Non-canonical FLT3 mutations

In recent years, an increasing number of non-ITD and D835 mutations in *FLT3* have been described [9–11]. The landscape and frequency of these non-canonical (NC) *FLT3* mutations are challenging to assess, as they are not detected using standard-of-care PCR-based assays. Estimated prevalence from next-generation sequencing (NGS)-based studies is on the order of 5–8%. In a large whole genome sequencing study of 799 pediatric patients with AML, 7.6% of patients harbored NC *FLT3* mutations, 9 of which were JMD mutations [12]. Similarly, in a recent study using high-throughput genomic sequencing of patients treated on the RATIFY trial, 26/275 (5.5%) of patients harbored NC *FLT3* mutations concurrent with either *FLT3*-ITD or TKD mutations [13].

To date, most NC *FLT3* mutations have been described in either the JMD or TKD [9, 10, 12], although NC FLT3 mutations have been described in the extracellular domain as well [14]. Of the JMD and KD mutations described in patients with functional correlates, most demonstrate increased signaling activation or autophosphorylation [10, 15–17]**.** In a recent study of NC JMD FLT3 deletion mutations, the Catalogue of Somatic Mutations in Cancer (COSMIC) database was queried for deletions observed in patients spanning *FLT3* residues 572–575 [10]. Mutations at four residues were identified (Y572, E573, S574, Q575), and when these deletions were introduced into cell lines, all demonstrated FLT3 autophosphorylation and increased downstream signaling [10]. This suggests that at least some NC *FLT3* mutations are driver mutations, actively promoting leukemic proliferation and survival. Consistent with this, another recent study of JMD deletion mutations reported sensitivity to FLT3 inhibition [18]. As sequencing technologies improve and broad NGS panels are increasingly used, the full landscape of NC FLT3 mutations observed in AML patients and their prognostic significance will become more apparent.

## FLT3 clonal architecture, selection, and evolution

AML is characterized by a complex polyclonal architecture and evolution. While 30% of patients with newly diagnosed AML harbor a canonical FLT3 mutation, many patients will additionally develop a de novo FLT3 mutation after treatment. Pre-existing FLT3-mutated clones can also expand under therapeutic pressure.

Historically, fit patients with *FLT3-*mutated AML were treated with cytotoxic chemotherapy alone. Although patients with *FLT3-ITD* mutated disease frequently responded to chemotherapy at rates similar to patients with *FLT3*-WT disease, responses were short-lived, with rapid outgrowth of the *FLT3* mutated clone [19]. Along with cytotoxic chemotherapy, selection for *FLT3*-mutated clones is a common resistance mechanism to venetoclax-based therapies which are the current standard-of-care for older or unfit patients with newly-diagnosed AML [20, 21]. In an analysis of 81 older or unfit patients treated with venetoclax doublets, expansion or acquisition of *FLT3*-mutant clones was the second-most common adaptive resistance mechanisms, second only to biallelic *TP53* mutations [20].

*FLT3* clonal selection also drives resistance to other targeted therapies [22–24]. In a study of 174 patients treated with the isocitrate dehydrogenase 1 (IDH1) inhibitor ivosidenib, pre-treatment presence of a *FLT3* mutation was associated with a significantly lower likelihood of response, and both *FLT3*-ITD and TKD mutations commonly emerged on relapse [22]. In a longitudinal analysis of 60 patients treated with IDH1 or 2 inhibitors, no patients with concurrent *FLT3* mutations responded to IDH inhibition, although this was not statistically significant due to the small sample size [23].

## Targeting FLT3 today

Despite the therapeutic resistance of *FLT3*-mutated AML, AML blasts harboring *FLT3*-ITD and TKD mutations are sensitive to small molecule inhibitors; as such, these targeted FLT3 inhibitors are an important part of the therapeutic approach to treating patients with *FLT3*-mutated AML.

### Newly diagnosed disease

#### Fit patients

For newly diagnosed, fit patients with *FLT3-*mutated AML, the standard of care is defined by the phase III RATIFY trial, in which patients with *FLT3-*ITD or -TKD mutated AML were randomized to standard induction and consolidation chemotherapy ± midostaurin [25]. The addition of midostaurin resulted in superior overall survival (OS) compared to chemotherapy alone (median OS 74.7 vs 25.6 months, *p = *0.0009) [25]. Based on these results, in 2019, midostaurin became the first approved agent for *FLT3-*mutated AML. Although RATIFY only included patients through age 60, the recent AMLSG 16–10 trial compared midostaurin plus chemotherapy against historical outcomes in fit adults aged 61–70 and found midostaurin-containing therapy to be superior as well [26]. Midostaurin has also been evaluated in combination with liposomal daunorubicin and cytarabine (CPX-351) in newly diagnosed adults with *FLT3-*mutated AML up to age 75; preliminary results of 23 patients indicate this regimen is safe and efficacious, with a complete response (CR) rate of 82% [27].

More recently, the phase III QuANTUM-First trial randomized 539 patients ages 18–75 with *FLT3-*ITD mutated AML to induction and consolidation chemotherapy plus quizartinib vs placebo; median overall survival was superior in the quizartinib arm (31.9 vs 15.1 months, *p = *0.032) [28]**.** Unlike RATIFY, QuANTUM-FIRST included an assessment of the depth of response measured by measurable residual disease (MRD). In this assessment, the proportion of patients with composite complete remission with *FLT3*-ITD MRD less than 10^−4^ was similar across groups (25% for quizartinib versus 21% for placebo) but the proportion of patients with composite complete remission with undetectable MRD was higher for quizartinib (14% versus 7%), suggesting increased depth of response. Based on these results**,** quizartinib recently received approval in Japan for newly diagnosed *FLT3*-ITD mutant AML and is currently undergoing similar regulatory consideration by the U.S. Food and Drug Administration (FDA). As quizartinib and midostaurin-containing regimens have not been directly compared, it is unclear how quizartinib will factor into current treatment paradigms for *FLT3*-ITD + patients, though midostaurin should remain the standard of care for patients with *FLT3* TKD mutations. Additional phase III frontline trials of cytotoxic chemotherapy with crenolanib (NCT03258931) and gilteritinib (NCT03936209; HOVON 156 AML/AMLSG 28–18) vs midostaurin are ongoing. Importantly, similar to QuANTUM-First, these ongoing trials incorporate MRD monitoring, providing key information as to how well these regimens suppress the *FLT3-*mutant allele and whether depth of response correlates with outcome.

#### Unfit patients

For older and/or unfit patients, venetoclax plus hypomethylating agents (HMA) are the standard of care. While the first-generation FLT3 inhibitor sorafenib plus azacitidine is an approved regimen per the National Comprehensive Cancer Network (NCCN) guidelines [28], it does not appear to outperform approved venetoclax-based regimens in this population. In a phase II trial of sorafenib plus azacitidine in newly diagnosed older adults with AML, the overall response rate (ORR) was 78% with an OS was 8.3 months [29]. By contrast, in older/unfit patients with newly diagnosed, *FLT3-*mutated AML treated with HMA plus venetoclax, the ORR was 70% with a median OS of 15 months in subgroup analysis [30].

### Relapsed or refractory disease

For both fit and unfit patients, the current standard of care for relapsed or refractory (R/R) *FLT3*-mutated AML in the U.S. and Europe is single-agent gilteritinib; in Japan, single-agent quizartinib is also an option. The phase III ADMIRAL trial compared gilteritinib vs salvage chemotherapy; gilteritinib demonstrated a greater CR rate (34 vs 15%, *p = *0.0001) and superior median OS (9.3 vs 5.6 months, *p* < 0.0001) [30]. Similarly, in the phase III QUANTUM-R trial, single-agent quizartinib was associated with superior OS compared to salvage chemotherapy (6.2 vs 4.7 months, *p = *0.02) [31]. Quizartinib is now approved in Japan, although concerns about study design led to both FDA and EMA rejection [31].

### Maintenance

#### Post-transplant

Consolidation with allogeneic hematopoietic cell transplantation (HCT) is currently recommended in eligible patients [32]. Following HCT, maintenance therapy with various FLT3 inhibitors has been studied. In the phase II RADIUS trial, FLT3-ITD + patients in the first CR were randomized to midostaurin vs placebo maintenance for 12 months; there was no difference in relapse-free survival [33]. In the phase II SORMAIN trial, patients with *FLT3-*ITD-mutated AML in remission after HCT were randomized to sorafenib vs placebo. Sorafenib maintenance resulted in superior 2-year estimated probability of survival (90.5% vs 66.2%, *p = *0.007), although the study was terminated early after not reaching the targeted accrual [34]. In a subsequent phase III study, 202 patients were randomized to sorafenib maintenance vs standard care post-HCT, and sorafenib resulted in fewer relapses at 1-year post-HCT (7% vs 24.5%, *p = *0.001) [35]. Of note, in both trials, only 21% and 24% of patients had received an FLT3 inhibitor prior to HCT and both included patients in first and second CR. It is unknown whether the benefit of sorafenib applies to patients transplanted in the first CR after standard-of-care frontline therapy [34, 35]. More recently, the phase III MORPHO trial randomized patients to gilteritinib vs placebo following HCT for patients transplanted in the first CR, and indicated no overall difference in RFS for patients treated with gilteritinib [36]. However, in the 50.6% of patients with MRD positivity (detected using PCR NGS at sensitivity of 10^–6^ or greater), the effect of gilteritinib on RFS was pronounced (HR = 0.515, 95% CI: 0.316, 0.838, *p = *0.0065) compared to patients without detectable MRD (HR = 1.213, 95% CI: 0.616, 2.387, *p = *0.575) [37]. These data indicate a benefit of gilteritinib maintenance for patients with MRD transplanted in CR1.

#### Post-chemotherapy

For patients who are either ineligible for or do not proceed directly to transplant, maintenance FLT3 inhibitor therapy can be considered. While RATIFY did allow for midostaurin monotherapy after consolidation and is approved as such in Europe, midostaurin maintenance was not efficacious in post hoc analysis and approval was not extended by the FDA [38]. Similarly, in the SORAML trial of sorafenib vs placebo plus chemotherapy in newly diagnosed FLT3 mutated AML, patients randomized to the sorafenib arm received post-chemotherapy sorafenib maintenance. While the RFS curves did separate throughout the maintenance phase, the trial was not powered to detect whether maintenance sorafenib contributed to improved outcomes [39].

Multiple studies of maintenance FLT3 inhibitors are ongoing. In the recently completed QuANTUM-First trial, patients randomized to the quizartinib arm received both post-chemotherapy and/or post-HCT quizartinib maintenance; report of these outcomes is anticipated in future publications [28]. Similarly, ongoing frontline trials of crenolanib vs midostaurin and gilteritinib in combination with chemotherapy will include crenolanib and gilteritinib maintenance, respectively (NCT03258931, NCT03936209, HOVON 156 AML/AMLSG 28-18).

## Resistance to FLT3 inhibitors

**Fig. 1:**
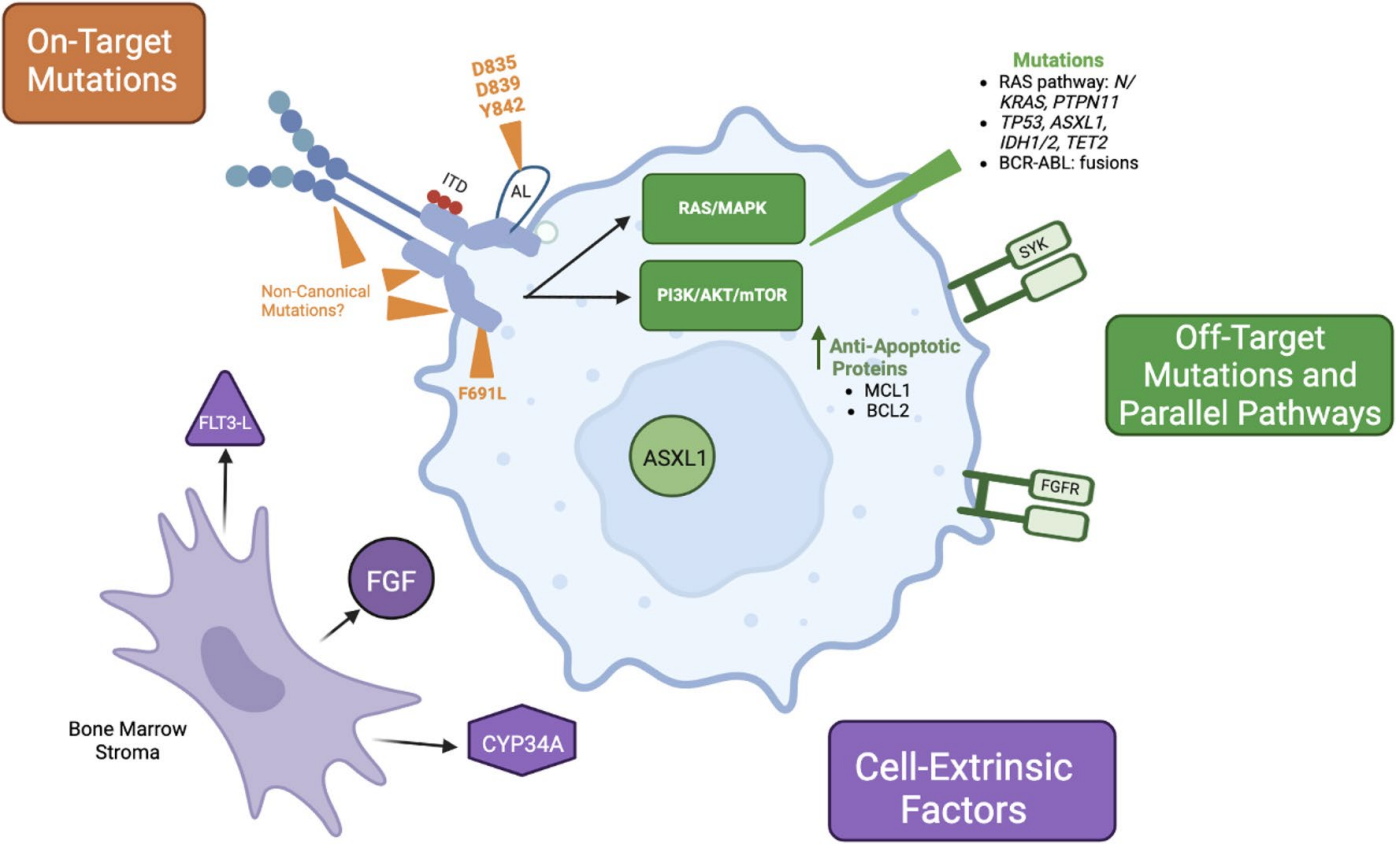
Described mechanisms of cell-intrinsic and extrinsic FLT3 inhibitor resistance mechanisms, including on-target secondary mutations within *FLT3*, off-target mutations in parallel and/or downstream pathways, upregulation of antiapoptotic proteins, and factors upregulated in the bone marrow microenvironment

**Fig. 2:**
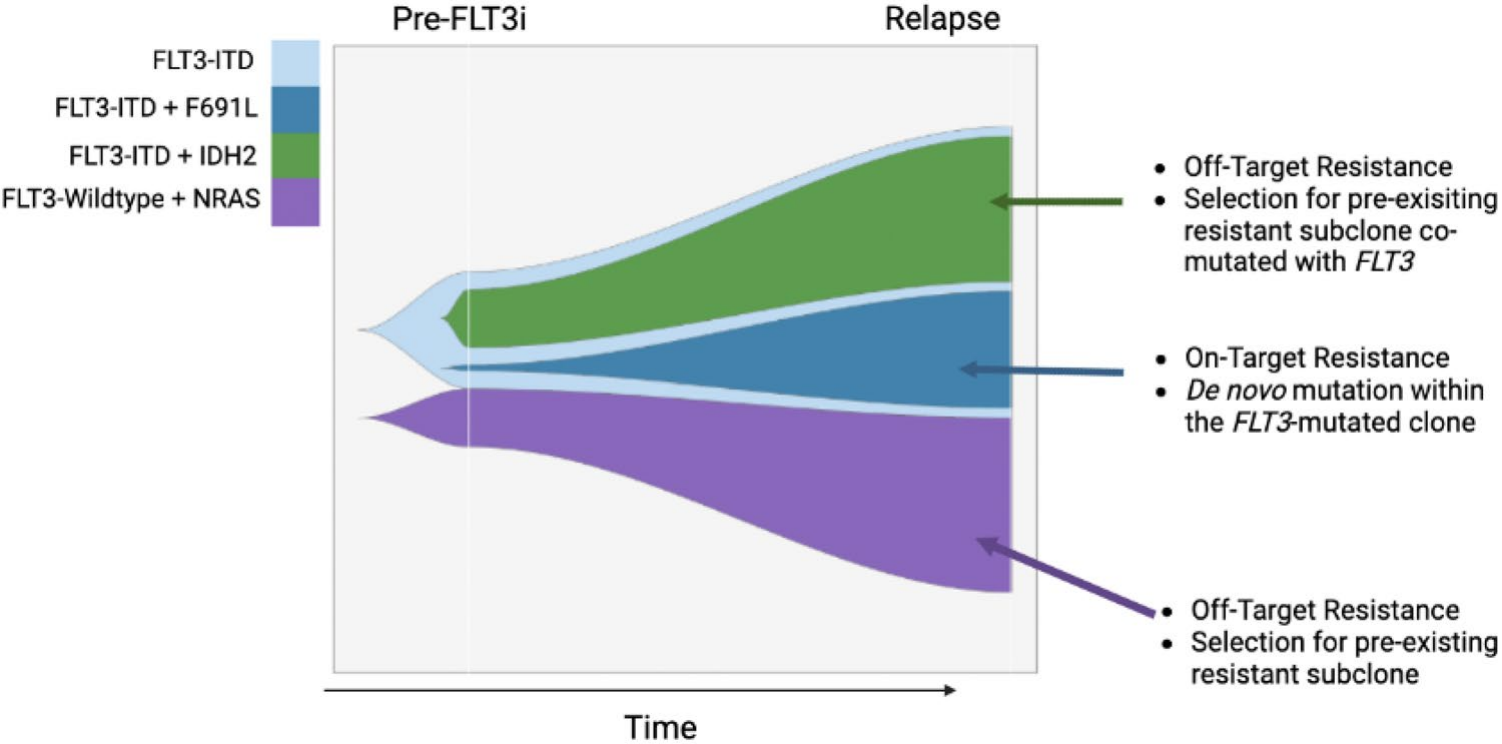
Fish plot highlighting complex clonal selection with FLT3 inhibitor resistance in an imagined patient. The green, blue, and purple portions of the plot represent clones with *FLT3-ITD* + *IDH2, FLT3-ITD* + *FLT3 F691L,* and *NRAS* genotypes, respectively. Over time and with FLT3 inhibitor resistance, all three of these clones expand

Although FLT3 inhibitors have significantly improved the survival of patients with *FLT3*-mutated AML, resistance remains an ongoing challenge. Resistance mechanisms to FLT3 inhibitors are heterogenous and comprise both cell intrinsic and extrinsic processes (Fig. 1) as well as complex clonal selection and evolution (Fig. 2).

### Genetic resistance: on-target secondary mutations

One key resistance mechanism to FLT3 inhibition is the development of on-target mutations within the *FLT3* gene. In many cases, these secondary mutations are not detected prior to FLT3 inhibitor treatment and are instead presumed to evolve de novo, or at least below the detection limit of current sequencing technologies [40, 41]. In type II FLT3 inhibitor therapy, on target resistance is frequently medicated by *FLT3*-TKD mutations, most commonly at D835 although mutations in kinase domain residues I836, D839, and Y842 have been described as well [41]. Less commonly, on target resistance can occur via development of the “gatekeeper” mutation F691L, which confers resistance to both type I and type II FLT3 inhibitors [42]. Practically, the development of F691L mutations likely represents a minority of gilteritinib-resistant leukemias. In studies profiling mutations in patients who relapsed following gilteritinib monotherapy on the ADMIRAL trial, only 5/4 (12.5%) of evaluable patients developed F691L mutations at relapse [40, 43].

As sequencing technologies improve, an increasing number of NC *FLT3* mutations are described in on-target resistance. Multiple in vitro studies have now demonstrated variable FLT3 inhibitor resistance mediated by NC *FLT3* mutations [44–46]**.** The resistance caused by these NC mutations may be unique to individual inhibitors. For example, one in vitro study of co-mutated *FLT3* N701K plus *FLT3-*ITD cells demonstrated resistance to type I inhibitor to gilteritinib and sensitivity to type II inhibitor quizartinib, a pattern opposite to that seen in *FLT3*-TKD on-target resistance mutations [44]. Data describing whether and to what extent NC *FLT3* mutations convey resistance in patients is more limited. In the same analysis of 40 patients with *FLT3*-mutated AML treated with gilteritinib monotherapy on the ADMIRAL trial, 2 patients developed de novo NC JMD mutations at E598D [40]. Similarly, the development of JMD mutation at Q575 has been described in a patient resistant to cytotoxic chemotherapy plus gilteritinib [10], and the development of KD mutation at N676 has been described in several patients with resistance to midostaurin [44, 47]. While these case studies are intriguing, full sequencing of the *FLT3* gene is needed in correlative analyses of future trials to fully understand the impact of the NC *FLT3* mutations on disease resistance.

### Genetic resistance: off-target mutations

A second mechanism of cell-intrinsic FLT3 inhibitor resistance is the emergence or expansion of clones with mutations outside of *FLT3*. These off-target mutations are heterogenous, including genes involved in Ras/MAPK signaling (*NRAS, KRAS, PTPN11*) as well as *ASXL1*, *TP53, TET2, IDH1/2* [14, 40, 43, 48]*.* Development of *BCR-ABL1* fusion genes with gilteritinib resistance has been described as well [43, 49]. In a study of paired pre-treatment and relapse samples from patients treated on the RATIFY trial, at relapse, almost half of patients (46%) became *FLT3*-negative but acquired mutations in other signaling pathways, indicating a strong selective advantage for these clones [48].

Of these heterogenous off-target resistance mechanisms, mutations activating the downstream Ras/MAPK pathways are particularly common, especially in resistance to type I inhibitors gilteritinib or crenolanib [14, 40, 43]**.** In patients who relapsed following gilteritinib monotherapy, new mutations in Ras/MAPK pathway genes occurred in 45% of patients, nearly triple the observed number of new on-target mutations [40]. In a similar study of 41 patients treated with single-agent gilteritinib, treatment-emergent Ras/MAPK mutations were identified in 37%; of these, over half had multiple *RAS* mutations [43]**.** Finally, in a recent analysis of 12 patients treated with the combination of gilteritinib and venetoclax, 4/12 patients developed dominate *N*/*KRAS* mutations at relapse; notably, no patients developed on-target mutations [50]. While best described in resistance to type II inhibitors, *Ras/MAPK* pathway mutations may be an important resistance mechanism to type II inhibitors as well. In an analysis of 8 patients with relapsed disease after quizartinib monotherapy, 2/8 patients demonstrated outgrowth of *N/KRAS* mutations, although on-target mutations were the dominate resistance mechanism in this cohort, present in 7/8 patients [41].

Recently, single-cell sequencing studies have shed light on the clonal architecture of these off-target mutations. While off-target mutations can occur in *FLT3-*mutant blasts, they frequently occur in cells without co-occurring *FLT3* mutations and are often present prior to FLT3 inhibitor therapy [14, 41, 43, 50]**.** In a study of 12 patients treated with gilteritinib and venetoclax and analyzed by single-cell sequencing, all 4 patients with dominate *N/KRAS* mutations at relapse had mutations detected prior to therapy [50]. This suggests that resistance via off-target mutations frequently arises via selection of pre-existing subclones which harbor a survival advantage with FLT3 inhibition, a mechanism distinct from that seen in the development of de novo on-target secondary mutations.

### Non-genetic resistance

Non-genetic mechanisms are also potential key mediators of FLT3 inhibitor resistance. In a study of 40 patients relapsed after gilteritinib with paired pre- and post-treatment samples, 13/40 patients had no new detectable mutations detected at relapse, suggesting that non-genetic mechanisms may be driving resistance in a significant proportion of patients [40].

Multiple components of the bone marrow microenvironment may facilitate FLT3 inhibitor resistance. The bone marrow microenviroment harbors FLT3 ligand, and levels may be particularly increased during induction or consolidation chemotherapy [51, 52]. Although FLT3 inhibitors effectively target *FLT3* mutations, they are less effective at disrupting wild-type FLT3 signaling, and high FLT3 ligand concentrations are protective against FLT3 inhibitors [51, 53]. Bone marrow stromal cells also have high levels of CYP34A expression, leading to increased FLT3 inhibitor metabolism [54]. Paralleling the observation that off-target mutations in downstream pathways are key in cell-intrinsic resistance, bone marrow stromal cells can also directly upregulate Ras/MAPK signaling via FGF2, activate STAT5 signaling, and inhibit blasts apoptosis via activation of the ATM/mTOR pathway [55–57]. Together, these alterations in the bone marrow microenviroment may be protective of residual leukemia and contribute to early disease resistance relative to genetic resistance mechanisms [58].

Finally, emerging evidence suggests leukemic differentiation state may also contribute to FLT3 inhibitor resistance. In a large study of ex vivo drug sensitivity testing of samples from 279 patients with newly diagnosed AML, monocytic cell state was associated with increased resistance to sorafenib, independent of other genetic alterations [59]. In a multi-omic single-cell sequencing analysis of 12 patients with resistance to gilteritinib and venetoclax, multiple subclones of heterogenous genotypes demonstrated increased expression of monocytic markers with therapy resistance [50]. Whether monocytic cell state is truly an independent resistance mechanism to FLT3 inhibitors or a bystander to other cell-intrinsic or extrinsic resistance mechanisms is unknown.

## Targeting *FLT3* today: FLT3 combination therapies

Identifying strategies to overcome resistance and extend disease response is an area of active research. Given the broad array of resistance mechanisms to FLT3 inhibition, as well as the polyclonal nature of *FLT3-*mutated AML, one of the most promising current strategies is to combine FLT3 inhibitors with other antileukemic agents (Tables 1, 2).

**Table 1:** Select complete and ongoing trials of FLT3 inhibitor doublet combinations

Agents studied	Phase	Treatment setting	Patient population	Results (if available)
HMA-based doublets				
Sorafenib + azacitidine	II	• ND and not eligible for intensive therapy	• *n* = 27 • *FLT3*-ITD • Age: median 74 (61–86)	• Primary endpoint: ORR 78% • Median response duration: 14.5 months • Median OS: 8.3 months
Sorafenib + azaciditine	I/II	• ND and not eligible for intensive therapy • R/R	• *n* = 37 (6 ND; 31 R/R) • *FLT3-*ITD (93% of patients) • Age: median 64 (24–87)	• Primary endpoint: ORR 46% (16% CR, 27% CRi, 3% SD)
Quizartinib + azacitidine OR low-dose cytarabine	I/II	• ND and not eligible for intensive therapy • First salvage	• *n* = 73 (34 ND; 39 first salvage) • *FLT3*-ITD • Age: median 72 (52–82) [ND]; 65 (24–84) [first salvage]	• Primary endpoint: cCR rate 87% (newly diagnosed); 64% (first salvage) • Median OS 19.2 months (newly diagnosed), 12.8 months (first salvage)
Gilteritinib + azacitidine vs. azacitidine alone	III	• ND and not eligible for intensive therapy	• *n* = 123 • *FLT3*-ITD or TKD • Age: median 78 (59–90) [gilteritinib arm]; 76 (61–88)	• Primary endpoint: Median OS 9.8 months [gilteritinib arm] vs 8.9 months, not significant
Ponatinib + azacitidine	Ib	• ND and not eligible for intensive therapy • R/R	• *n* = 31 • *FLT3-*ITD (90%) or *CBL* (10%) • Age: median 67 (26–87)	• ORR: 52% • Median duration of response: 12.9 months
Venetoclax-based doublets				
Gilteritinib + venetoclax	Ib	• R/R	• *n* = 61 • *FLT3-*ITD (72%), TKD (15%), both (5%) • Age: median 63 (21–85)	• cCR rate 75% • Median duration of response: 4.9 months • Median OS: 10 months
Quizartinib + venetoclax	Ib/II	• R/R	• *FLT3*-ITD • Age > = 18	Trial Ongoing: NCT03735875

*ND* newly diagnosed, *R/R* relapsed/refractory, *ORR* overall response rate, *CR* complete response, *CRi* CR with incomplete hematologic recovery, *SD* stable disease, *cCR* composite *CR*, *OS* overall survival

**Table 2:** Select active trials of FLT3 inhibitor triplet combinations

Agents studied	NCT	Phase	Treatment Setting	Patient Population	Results (if available)
Quizartinib + decitabine + venetoclax	NCT03661307	I/II	• R/R	• *FLT3*-ITD • age ≥ 18	• Interim cRC rate: 82% (CR 11%, CRi 28%, MLFS 43%)
Gilteritinib + oral decitabine plus cedazuridine + venetoclax	NCT05010122	I/II	• R/R	• *FLT3*-ITD or TKD • age 18–75	• N/A
Gilteritinib + azacitidine + venetoclax	NCT05520567	I/II	• ND and not eligible for intensive therapy • R/R	• *FLT3*-ITD or TKD • age ≥ 18	• Interim cRC rate 100% (95% CR, 5% MLFS) [ND]; 74% (21% CR, 16% CRi, 37% MLFS) R/R
Midostaurin + low-dose cytarabine + venetoclax	N/A	Ib/II	• ND and not eligible for intensive therapy	• All AML (*FLT3*-mut and wildtype) • Age ≥ 60	• Interim cRC rate 77.8% (not stratified by *FLT3* mutation status)
Investigator’s choice FLT3i + decitabine + venetoclax	NCT03404193	II	• ND and not eligible for intensive therapy • R/R	• All AML; addition of FLT3i per investigator’s discretion • Age ≥ 18 [ND], ≥ 60 [R/R]	• Interim cRC rate 92% [ND]; 62% [R/R]

*ND* newly diagnosed, *R/R* relapsed/refractory, *CR* complete response, *CRi* complete response with incomplete hematologic recovery, *MLFS* morphologic leukemia-free state, *cCR* composite CR

### Hypomethylating agents

Aside from conventional cytotoxic chemotherapy, one of the earliest FLT3 inhibitor combinations was with hypomethylating agents (HMA) azacitidine or decitabine. In a phase II trial of sorafenib plus azacitidine in newly diagnosed older adults with *FLT3-*ITD mutated AML, the ORR rate was 78% with an OS of 8.3 months [29]. In a similar trial of patients with R/R *FLT3-*ITD mutated disease, sorafenib plus azacitidine demonstrated an ORR 46% [60].

It is unclear whether combinations with the more potent second-generation FLT3i are more promising. In a phase I/II trial of patients with *FLT3-*ITD mutated AML treated with quizartinib plus azacitidine, the CR rate and median OS were promising at 87% and 19.2 months and 64% and 12.8 months in the frontline and R/R settings, respectively [61]**.** By contrast, in the recent randomized phase III LACEWING trial of older/unfit adults with newly diagnosed *FLT3-*mutated AML, there was no difference in OS for gilteritinib plus azacitidine vs azacitidine alone (9.8 vs 8.9 months), although these results were clouded by the fact that many patients on the azacitidine arm terminated the study early and received subsequent FLT3 inhibitor therapy [62]. Finally, the multi-kinase inhibitor ponatinib in combination with azacitidine demonstrated a durable ORR of 52% in unfit newly diagnosed patients with *FLT3*-mutated AML [63, 64].

### Venetoclax

Venetoclax, an oral inhibitor of the anti-apoptotic protein BCL-2, is particularly promising in combination with FLT3 inhibitors. Upregulation of antiapoptotic proteins is a mechanism of FLT3 inhibitor resistance [65], and the emergence or outgrowth of *FLT3* mutations is associated with venetoclax resistance [20, 66]**.** In vitro studies have demonstrated synthetic lethality with venetoclax combined with multiple FLT3 inhibitors [67–69]**.** In a phase Ib trial of 61 patients with R/R *FLT-*mutated AML treated with gilteritinib plus venetoclax, the modified CR rate was 75% with a median OS of 10 months [70]. This is substantially higher than the 54% modified CR rate observed in the ADMIRAL trial of single-agent gilteritinib using identical response criteria [71]. A trial of quizartinib plus venetoclax is ongoing (NCT03735875).

### Triplet combinations

Given the promising outcomes of both HMA and venetoclax doublet combinations, as well as the efficacy of HMA and venetoclax combinatory therapy, it is not surprising that triplet combinations have received particular attention. In a retrospective analysis of 87 newly diagnosed patients with *FLT3*-mutated AML treated with either triplet (HMA + Venetoclax + FLT3i) or doublet (HMA + FLT3i) therapy, patients receiving triplet therapy had significantly longer OS without increased cytopenias [72]. Intriguingly, while patients receiving doublet therapy still demonstrated a benefit from allogeneic transplant, survival for those receiving triplet therapy was similar irrespective of transplant status, suggesting a benefit of triplet therapy in the upfront setting may lay in a potential to forgo transplant.

In phase I/II trial of 28 patients with R/R *FLT3-ITD* mutated AML with prior FLT3 inhibitor treatment, the triplet quizartinib, decitabine, and venetoclax showed promising interim results, with a composite CR (cCR) rate of 82% [73]. Furthermore, in a smaller cohort of 7 newly-diagnosed patients treated with the same regimen, all patients achieved a cCR [73]. A trial of an all-oral version of the same regimen, using oral decitabine plus ceduazurdine (ASTX727), is ongoing [74]**.** In a phase I/II study of gilteritinib, azacitidine, and venetoclax in a similar patient population, 100% of newly diagnosed patients and 74% of patients with R/R disease achieved composite CR [75]**.** While these combinations have encouraging outcomes, both triplets had high incidence of myelosuppression, with a median time of both neutrophil and platelet recovery of over a month [73, 75]. Further data is needed to understand how to maximize the safety of these regimens, particularly in older and/or frailer patients, as well as how these regimens compare to HMA plus venetoclax or FLT3 inhibitor plus venetoclax doublet therapies, especially in R/R patients where response rates to FLT3i/venetoclax doublets and triplets are similar. [76, 77]

Will these combinatory therapies be able to fully overcome established resistance mechanisms? Thus far, it appears unlikely. For example, in patients who relapsed after single-agent gilteritinib in the ADMIRAL trial, the most common mutations associated with treatment resistance were in genes associated with the Ras/MAPK pathway [40]. Similarly, mutations in *NRAS* and *KRAS* appear to be the dominant genetic resistance mechanism to gilteritinib plus venetoclax [50], and patients with Ras/MAPK mutations had the lowest response rate to the triplet quizartinib/decitabine/venetoclax [73]. Although inhibitors to Ras/MAPK signaling, including *BRAF* V600E and *KRAS* G12C inhibitors, are approved for other cancers, the Ras/MAPK pathway mutations observed in resistance to FLT3 inhibitors are not sensitive to these agents [78, 79]. New targeted therapies, either in combination with or sequential to FLT3 inhibitors will be needed to overcome these dominant resistance mechanisms.

## Novel FLT3 targeting approaches

In addition to established combination partners like HMA or venetoclax, FLT3 inhibitors have also been combined with novel small molecules, including the spleen tyrosine kinase inhibitor lanraplenib, lysine-specific demethylase-1 inhibitor iadademstat, and approved IDH1/2 inhibitors ivosidenib and enasidenib [80, 81]. Studies involving multikinase inhibitors, such as the FLT3/SYK/JAK/KIT kinase inhibitor tuspetinib [82], the FLT3/BTK inhibitor luxeptinib [83], and the FLT3/FGFR inhibitor MAX-40279 [84] are ongoing as well. In addition to these agents, novel covalent FLT3 inhibitors and FLT3-targeted biologic agents are in active pre-clinical and clinical development as well [85, 86] (Table 3).

**Table 3:** Select active trials of novel FLT3 inhibitor combinations, multikinase inhibitors, and biologic therapies

Agent(s) studied	Novel mechanism	NCT	Phase	Treatment setting	Patient population
Novel combination partners					
gilteritinib + lanraplenib	Lanraplenib: Spleen Tyrosine Kinase (SYK) inhibitor	NCT05028751	Ib/II	• R/R	• *FLT3*-ITD or TKD • Age ≥ 18
Gilteritinib + iadademstat	Iadedemstat: lysine-specific demethylase-1 inhibitor	NCT05546580	I	• R/R	• *FLT3*-ITD or TKD • Age ≥ 18
Gilteritinib + ivosidenib or enasidenib	Ivosidenib: IDH1 inhibitor; Enasidenib: IDH2 inhibitor	NCT05756777	Ib	• R/R • Morphologic remission with MRD persistence	• *FLT3-*ITD or TKD and concurrent *IDH1* or *IDH2* • Age ≥ 18
Multikinase inhibitors					
tuspetinib (HM43239) ± venetoclax (phase II portion only)	Inhibits FLT3, SYK JAK, KIT	NCT03850574	I/II	• R/R	• All AML • Age ≥ 18
Luxeptinib (CG-806)	Inhibits FLT3, BTK	NCT04477291	Ia/II	• R/R	• All AML, high-risk MDS • Age ≥ 18
MAX-40279	Inhibits FLT3, FGFR	NCT03412292	I	• R/R	• All AML • Age ≥ 18
Dubermatinib (TP-0903) + azacitidine	Inhibits FLT3, AXL	NCT04518345	I	• R/R	• *FLT3*-ITD • Age ≥ 18
Emavusertib (CA-4948) ± azacitidine ± venetoclax	Inhibits FLT3, IRAK4	NCT04278768	I	• R/R	• All AML, high-risk MDS with > 8% blasts
Biologic therapies					
TAA05	Anti-FLT3 CAR T Cell	NCT05445011	I	• R/R	• *FLT3*-ITD • Age 18–70
AMG 553	Anti-FLT3 CAR T Cell	NCT03904069	I	• RR	• All AML, but blasts must express FLT3 by flow cytometry • Age ≥ 12
CLN-049	FLT3 x CD3 bi-specific T cell engager	NCT05143996	I	• R/R	• All AML, high-risk MDS • Age ≥ 18

*R/R* relapsed/refractory, *MRD* measurable residual disease

## Prognosis of FLT3-mutated AML in the modern era

Historically, the presence of a high *FLT3*-ITD allelic ratio (AR), defined as a *FLT3*-ITD to *FLT3*-WT ratio of >  = 0.5, was associated with highest disease risk, *FLT3*-ITD AR < 0.5 with a co-occurring *NPM1* mutation was associated with favorable disease risks, and *FLT3-*ITD AR < 0.5 AR without a co-occurring *NPM1* mutation was associated with intermediate disease risk. This risk stratification was described in the 2017 European LeukemiaNet (ELN) schema [8]**.**

When these 2017 guidelines were developed, no FLT3 inhibitors were approved. Today, the paradigm has shifted considerably. Given this new treatment landscape, the prognostic significance of *FLT3* mutations has evolved. In an analysis of 513 patients with newly diagnosed AML, 96 patients with *FLT3-ITD* mutations experienced survival comparable with other patients with intermediate-risk features, and neither co-mutations in *NPM1* nor *FLT3*-*ITD* AR influenced outcomes [87]. Notably, in this cohort, only 41% of patients received an FLT3 inhibitor, so it is possible outcomes would be even better if all patients received current standard-of-care FLT3 inhibition [87]. In a retrospective analysis of the RATIFY trial, midostaurin plus chemotherapy significantly improved overall survival for all 2017 ELN risk groups, with similar OS probabilities for the midostaurin arm in both intermediate- and adverse-risk disease [88]. Based on these and other studies, as well as challenges in standardizing AR measurements across laboratories, *FLT3*-ITD AR and co-mutational status is no longer taken into consideration in the most recent 2022 ELN risk stratification schema [3]. Patients who are *FLT3*-ITD positive are classified as intermediate risk, irrespective of allelic ratio or concurrent mutations in *NPM1 *[3].

**Fig. 3:**
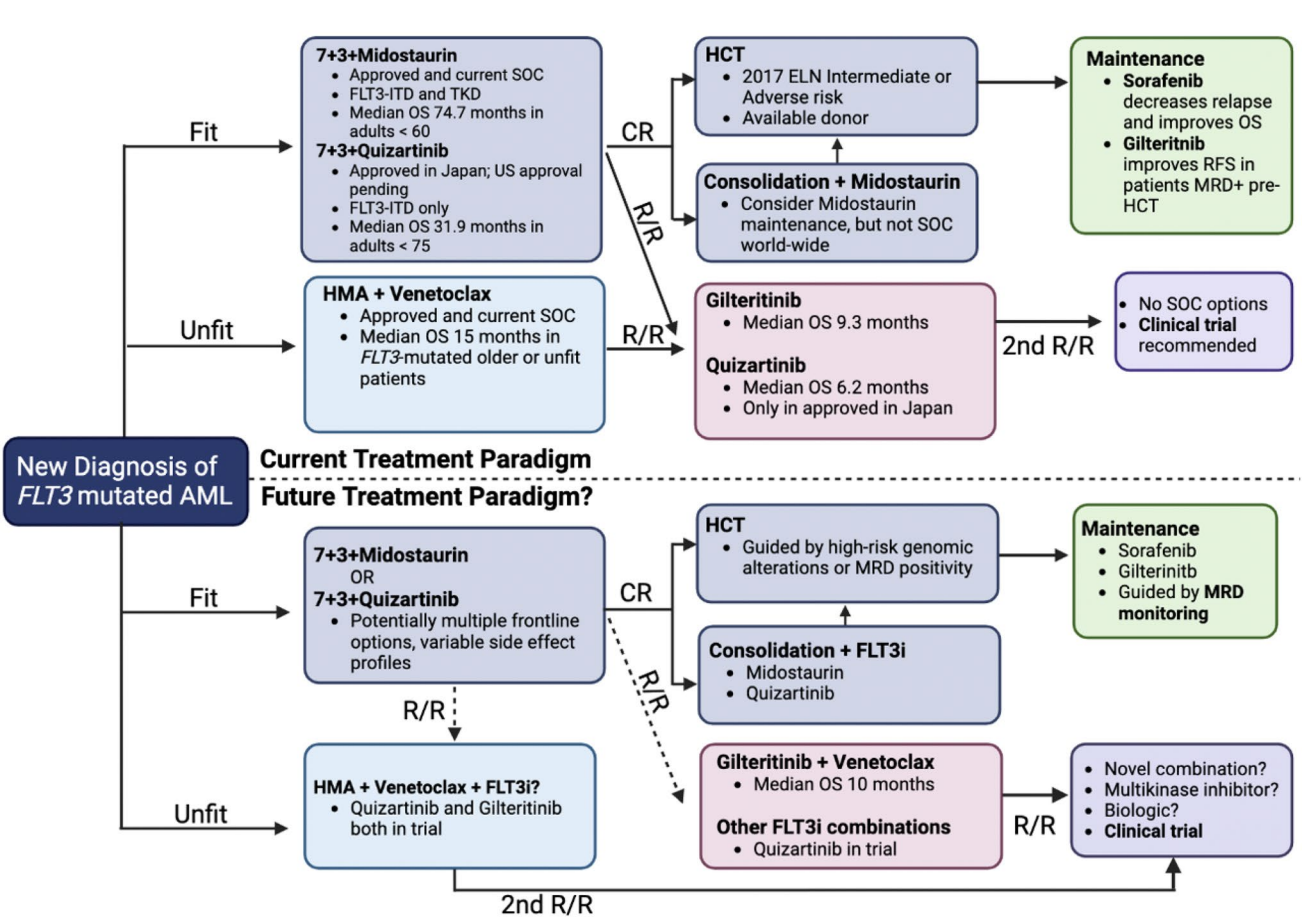
Schematic describing the current standard of care (top) and potential future treatment paradigm (bottom) of patients with *FLT3-*mutated AML. *SOC* standard of care, *OS* overall survival, *RFS* relapse-free survival, *MRD* measurable residual disease, *HCT* hematopoietic cell transplantation, *ELN* European LeukemiaNet

This evolving risk stratification and treatment landscape raises many questions. Now that all *FLT3-ITD* mutated AML is classified as intermediate risk, should all eligible patients with *FLT3-ITD* mutated AML receive an allogeneic hematopoietic stem cell transplant (HCT) in the first CR, a strategy most beneficial in patients at the highest risk of relapse, or can transplant be reserved for first relapse or MRD positive disease? In addition to flow cytometry-based MRD measurements, there is emerging evidence that pre-transplant *FLT3* DNA-NGS-based MRD can predict post-transplant outcomes [28, 37]. How will new therapies, such as quizartinib in newly diagnosed fit patients and FLT3 inhibitor/venetoclax-based combinations alter the current treatment paradigm and associated prognosis? Fig. 3 outlines possible directions for the treatment of *FLT3*-mutated AML in the future.

## Conclusion

Increasingly, treating FLT3-mutated AML represents the forefront of personalized medicine and targeted therapy in AML. As targeted FLT3 inhibitors and combinatory therapies become increasingly adopted, future risk stratification and treatment schema will evolve in tandem.
